# Modelling and Performance Analysis of MgB_2_ and Hybrid Magnetic Shields

**DOI:** 10.3390/ma15020667

**Published:** 2022-01-17

**Authors:** Michela Fracasso, Fedor Gömöry, Mykola Solovyov, Roberto Gerbaldo, Gianluca Ghigo, Francesco Laviano, Andrea Napolitano, Daniele Torsello, Laura Gozzelino

**Affiliations:** 1 Department of Applied Science and Technology, Politecnico di Torino, 10129 Torino, Italy; roberto.gerbaldo@polito.it (R.G.); gianluca.ghigo@polito.it (G.G.); francesco.laviano@polito.it (F.L.); andrea.napolitano@polito.it (A.N.); daniele.torsello@polito.it (D.T.); laura.gozzelino@polito.it (L.G.); 2 Istituto Nazionale di Fisica Nucleare, Sezione di Torino, 10129 Torino, Italy; 3 Institute of Electrical Engineering, Slovak Academy of Science, 84104 Bratislava, Slovakia; Fedor.Gomory@savba.sk (F.G.); mykola.solovyov@savba.sk (M.S.)

**Keywords:** magnetic shielding, superconductor modeling, MgB_2_ bulk superconductors

## Abstract

Superconductors are strategic materials for the fabrication of magnetic shields, and within this class, MgB${}_{2}$ has been proven to be a very promising option. However, a successful approach to produce devices with high shielding ability also requires the availability of suitable simulation tools guiding the optimization process. In this paper, we report on a 3D numerical model based on a vector potential (**A**)-formulation, exploited to investigate the properties of superconducting (SC) shielding structures with cylindrical symmetry and an aspect ratio of height to diameter approaching one. To this aim, we first explored the viability of this model by solving a benchmark problem and comparing the computation outputs with those obtained with the most used approach based on the **H**-formulation. This comparison evidenced the full agreement of the computation outcomes as well as the much better performance of the model based on the **A**-formulation in terms of computation time. Relying on this result, the latter model was exploited to predict the shielding properties of open and single capped MgB${}_{2}$ tubes with and without the superimposition of a ferromagnetic (FM) shield. This investigation highlighted that the addition of the FM shell is very efficient in increasing the shielding factors of the SC screen when the applied magnetic field is tilted with respect to the shield axis. This effect is already significant at low tilt angles and allows compensating the strong decrease in the shielding ability that affects the short tubular SC screens when the external field is applied out of their axis.

## 1. Introduction

Highly sensitive magnetic measurement systems, such as those employed in biomagnetic imaging or in other radiation/particle detection systems, need efficient magnetic shields to reduce the effects of the external magnetic disturbances [1,2,3,4,5]. This requirement is met with good results in a number of applications by superconducting (SC) devices. Recently, tested solutions have included both active (i.e., a set of coils fed with appropriate currents [6]) and passive layouts (i.e., a simple superconducting cavity [7,8,9]). The latter ones, which exploit the intrinsic property of the SC materials, can be assembled using SC bulks [10,11,12] and/or SC coated conductors/tapes [13,14,15,16,17]. Furthermore, promising improvements of the shielding ability have been achieved by superimposing SC and ferromagnetic (FM) materials [18,19,20,21,22,23,24], including the possibility to cloak static (DC) and alternating (AC) magnetic fields in suitably shaped SC/FM heterostructures [25,26,27,28,29,30].

In this framework, being able to model the electromagnetic behavior of an SC material as well as to guarantee high shielding factors for magnetic fields of arbitrary direction is a needed and successful approach for the development and optimization of novel screening devices [31]. Among the numerous numerical methods proposed to model the SC performances, the most widely used is the finite element method (FEM). The governing equations are Maxwell’s equations [32] and the superconducting material is usually described by means of a non-linear **E**–**J** characteristic, which takes into account the transition from superconducting to normal state [33]. This approach easily allows the implementation of the problem by self-developed programs or commercial software packages taking advantages of various formulations already developed.

In the last few years, the **H**-formulation has been the most commonly used approach. Actually, its implementation is quite simple since no gauging or post-processing is required [34]. However, using the **H**-formulation implies some challenges, as a higher number of degrees of freedom increases the computational time [35]. Furthermore, a degradation of the matrix conditioning is caused by the requirement of an artificial resistivity in non-conducting domains [34]. Nonetheless, different and mixed formulations such as **A**-ϕ, **H**-ϕ, **H**-**A**, **T**-**A**, **T**-ϕ, and **T**-**Ω** have been successfully implemented in electromagnetic dedicated FEM software, often in relation to a specific application [34,36,37,38,39,40].

In this work, an alternative approach based on a vector potential (**A**) formulation is employed to investigate the shielding properties of both superconducting and hybrid SC/FM screens in an applied magnetic field with several orientations. To this aim, this formulation, recently extended to solve 3D magnetic problems [41], was implemented in the commercial software COMSOL Multiphysics^®^ [42]. A preliminary validation of this modeling approach for shielding applications was already carried out via the comparison between experimental and computed data achieved on open and single-capped MgB${}_{2}$ tubes in both Axial Field (AF) and Transverse Field (TF) orientations [43,44]. Here, the model viability of evaluating the magnetic flux density values inside an SC tubular shield independently from the applied field orientation is addressed by comparing the results obtained with the **A**- and **H**-formulations. Since the aim is to assemble shielding shells that combine the practical requirement of a small size and a high shielding factor, we focused on MgB${}_{2}$ samples with a height/diameter aspect ratio close to unity. The material choice was driven by the MgB${}_{2}$ specific features, such as the low-cost, the use of non-toxic precursors (e.g., not containing rare earth elements), the low weight density, and the long coherence length. In particular, the last characteristic implies that boundaries among well connected grains do not prevent the flow of high current densities, thus opening to the employment of large polycrystalline manufacts—fabricated by in situ or ex situ sintering processes [45,46,47,48,49] or by infiltration processes [9,50,51]—for large scale applications.

The as-validated numerical procedure was then exploited to computationally investigate the role of a ferromagnetic layer in improving the screening ability of both open and single capped SC tubes (the latter henceforth named cups). Actually, in SC samples with cylindrical symmetry and such a small aspect-ratio, the effectiveness of the shielding properties strongly decreases when the field is not applied parallel to their axis [52]. On the other hand, tubular ferromagnetic shields are more efficient when the field is applied out of axis [53]. Therefore, the effects of the superimposition of an FM shell on the SC shields was investigated as a function of different magnetic field directions with respect to the shield symmetry axis.

The paper is organized as follows. Section 2 deals with the description of the samples and of the finite-element methods used for the numerical calculations. The shielding factors (SF) calculated using the **A**- and **H**-formulations for a benchmark geometry consisting in a short SC tube are compared in Section 3.1. In Section 3.2 and Section 3.3, the performances of SC and hybrid tube- and cup-shaped shields are investigated, respectively, as a function of the applied field orientation. The main outcomes are summarized in Section 4.

## 2. Materials And Methods

### 2.1. Materials Properties

All the superconducting shields were assumed to be made out of MgB${}_{2}$. The in-field behavior of the material was taken into account via the dependence of its critical current density $J_{c}$ on the magnetic field, as detailed in the next subsection. To this aim, we considered the critical current density values achieved at T=30 K from magnetic flux density measurements carried out on the axis of tube- and cup-shaped shields while the applied field was cycling [48,54]. These shields were obtained by carving fully machinable MgB${}_{2}$ cylinders, fabricated by spark plasma sintering of BN-added MgB${}_{2}$ powders [48,49]. We chose the working temperature of 30 K because it guarantees a negligible flux-jump occurrence, thus allowing the $J_{c}$ calculation [49], joined with still noteworthy shielding properties (by way of example, the cup shielding performances are still comparable with those found at 20 K on a YBa${}_{2}$Cu${}_{3}$O${}_{7}$ cup with similar aspect ratio [8]).

The FM shields were considered to be made out of Fe ARMCO with the same *B*–*H* constitutive law as the FM tube characterized experimentally in Ref. [21]. Fe ARMCO hysteresis losses are negligible; therefore, we did not account for them in modeling.

### 2.2. Basics of the **A**-Formulation

Relying on the numerical method presented by Campbell [55], Gömöry et al. [56] proposed an alternative 2D form of the critical state formulation using the vector potential **A** to model the behavior of SC materials. This approach was also successfully used to model SC samples in SC/FM hybrid structures [57]. Recently, Solovyov et al. [41] extended this formulation from 2D to 3D.

We implemented this numerical modeling by means of the Magnetic Fields interface (mf) of COMSOL Multiphysics^®^, which is suitable for computing magnetic fields and induced currents using Maxwell’s equations, expressed here in terms of the magnetic vector potential **A**. The superconducting behavior was described by means of the following non linear **E**–**J** relationship, which provides a smooth approximation of the critical state model function [56,58]:

(1) $$J=J_{c}\tanh\left(\frac{\left|E\right|}{E_{0}}\right)\left(\frac{E_{x}}{\left|E\right|}i+\frac{E_{y}}{\left|E\right|}j+\frac{E_{z}}{\left|E\right|}k\right)$$

where $\left(E_{x},E_{y},E_{z}\right)=\left(-\frac{\partial A_{x}}{\partial t},-\frac{\partial A_{y}}{\partial t},-\frac{\partial A_{z}}{\partial t}\right)$ is the local value of the electric field, and $J_{c}$ is the local critical current density $\left(J_{c}\geq \sqrt{J_{x}^{2}+J_{y}^{2}+J_{z}^{2}}\right)$. $E_{0}$ is a computation parameter that defines the sharpness of the transition function [41], which, in the case of power–law relation, is defined by the *n*-factor. Therefore, we would like to note that the physical meaning of this parameter is not the same as the criterion conventionally assumed to identify the value of the critical current density from the I–V experimental curves [33]. The chosen value $E_{0}=10^{-4}$ Vm${}^{-1}$ gives a good compromise between the computation performance and the accuracy for the presented superconducting object, considering the chosen range of applied magnetic field and its growth rate [44]. However, in case of other configurations it may require additional verification and adjustment of $E_{0}$ value.

Equation (1) ensures the collinearity between the current density and the electric field, expected in superconductors with isotropic properties, as the polycrystalline MgB${}_{2}$ bulks that we characterized are assumed to be [49]. This equation slightly differs from that reported in Ref. [41], whose formulation contained an imperfect isotropy for weak electrical fields. However, the results presented here were compared with those obtained using the previous formula, showing no discernible differences for this specific application of the model.

As we already reported in our previous works [43,54], a $J_{c}$ dependence on the magnetic field also needs to be taken into account. A previous experiment [48] evidenced that the experimental $J_{c}$ curves were successfully fitted by the following exponential relation:

(2) $$J_{c}\left(B\right)=J_{c,0}exp\left[-{\left(\frac{\left|B\right|}{B_{0}}\right)}^{\gamma }\right]$$

where $J_{c,0}$, $B_{0}$, and γ are constant parameters obtained for tube- and cup-shaped shields [48,54].

The source term for the applied magnetic field, $H_{appl}$, was considered through the boundary conditions: At a large distance from the shield(s), **B** was set to be equal to $\mu_{0}H_{appl}$. The applied field was always assumed uniform and increasing monotonically.

### 2.3. Basics of the **H**-Formulation

From a mathematical point of view, the **H**-formulation uses the finite-element method to solve Faraday’s equation, which in terms of the magnetic field **H** takes the form:

(3) $$\frac{\partial \mu_{0}\mu_{r}H}{\partial t}+\nabla \times \left(\rho \nabla \times H\right)=0$$

where $\mu_{r}$ is the relative magnetic permeability and ρ the resistivity [32]. We implemented the **H**-formulation by means of PDE module present in COMSOL Multiphysics^®^. The magnetic permeability was set to 1 in the whole space and the resistivity to $10^{8}\;\Omega$m in the air domain. Instead, the superconductor was modeled as a material with a non-linear electrical resistivity, showing the power–law dependence on the current density:

(4) $$\rho \left(J\right)=\frac{E_{0}}{J_{c}}{\left[\frac{\left|J\right|}{J_{c}}\right]}^{n-1}$$

where $E_{0}$ is the same conventional electric field as in Equation (1), $E_{0}=10^{-4}$ Vm${}^{-1}$, **J** is the current density, and $J_{c}$ is the critical current density. In this approach, the dependence of $J_{c}$ on the magnetic field was also taken into account by Equation (2) in the superconducting domain. The *n* value is a factor indicating the steepness of the transition from the superconducting to the normal state. According to [59], here, we set n=100.

The source term for the applied magnetic field was considered through the boundary conditions, as in the case of the **A**-formulation.

## 3. Results and Discussion

### 3.1. Solution of a Benchmark Problem via **A**- and **H**-Formulation Approaches

**Figure 1:**
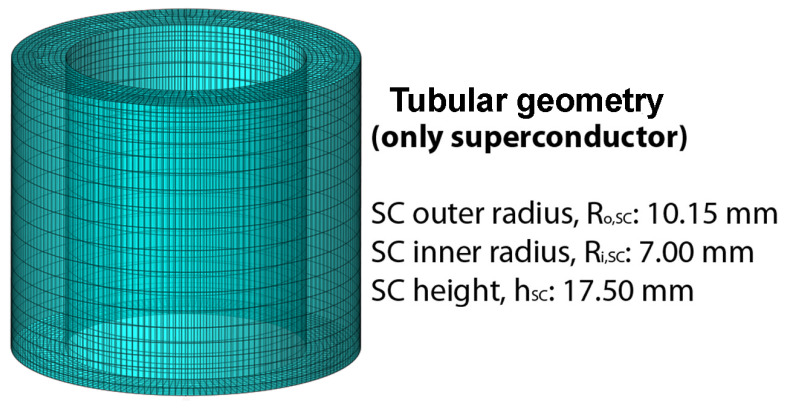
Schematic view of the SC shielding layout investigated with both the **A**- and **H**-formulations.

The comparison of the two numerical modeling approaches based on the **A**- and **H**-formulations was carried out investigating the shielding performances of the short hollow cylinder shown in Figure 1. Using this shape is very convenient from the computational time point of view and allows addressing the peculiarity of a short axisymmetric structure.

**Figure 2:**
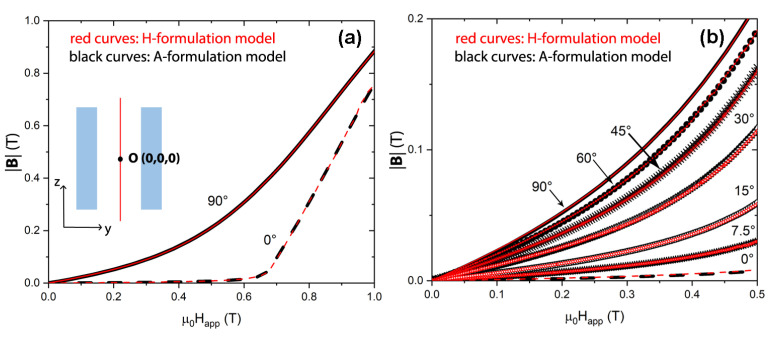
Comparison between the magnetic flux density values calculated in the tube center O (whose coordinates were set to (0,0,0)) of the tubular shield shown in Figure 1 using the **H**-formulation (red curves) and the **A**-formulation (black curves). Calculations were carried out in the axial and transverse field orientations (**a**) and for different angles of the applied field (**b**).

First, the study was carried out by applying the external field parallel and perpendicular to the tube axis, i.e., in the Axial Field (AF) and Transverse Field (TF) orientation, respectively, up to a maximum value $\mu_{0}H_{app}=1.7$ T, at which the superconductor is fully penetrated by the magnetic field. Then, focusing on the applied field range 0–0.5 T where the shielding effect is more significant, we calculated the magnetic flux density assuming the applied field tilted of 7.5°, 15°, 30°, 45°, and 60° with respect to the shield axis. To model the superconductor, the parameters $J_{c,0}=3.01\times 10^{8}$ A/m${}^{2}$, $B_{0}=0.83T$ and γ=2.52, experimentally found at *T* = 30 K for a similar MgB${}_{2}$ tubular shield [48], were employed in Equation (2). In Figure 2, the data calculated in the tube axis center O are shown.

As can be seen, the results obtained by means of the two different formulations exhibit the same behavior in the whole investigated range of field.

**Figure 3:**
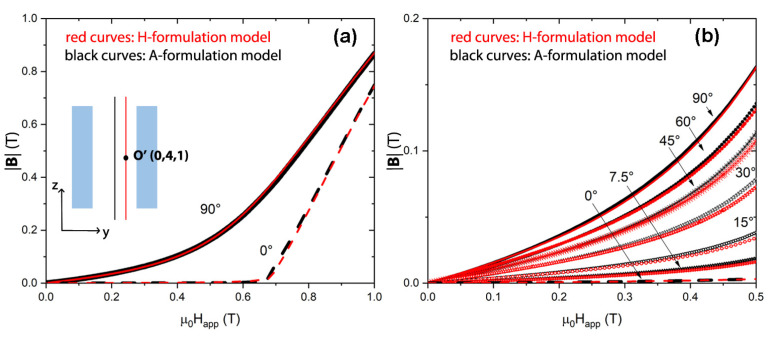
Comparison between the magnetic flux density values calculated at point O’ (whose coordinates related to point O are (0 mm, 4 mm, 1 mm), i.e., 4 mm away from the tube center along the radial direction and 1 mm away along the axial one), using the **H**-formulation (red curves) and the **A**-formulation (black curves). Calculations were carried out in the axial and transverse field orientations (**a**) and for different angles of the applied field (**b**).

Likewise, an excellent agreement was found by comparing magnetic flux density values calculated out of the tube axis. Figure 3 shows the **B** magnitude values found at position O’ placed 4 mm away from the center along the radial direction and 1 mm away along the axial direction. In this case, the magnetic flux density was also evaluated for the AF and TF orientations, as well as for the intermediate tilt angles of the applied magnetic field.

This comparison allows us to also validate the use of the proposed **A**-formulation model for magnetic shielding studies at different angles of the applied magnetic field. Indeed, it is worth mentioning that a first validation, limited to positions located along the shield axis and to the AF and TF orientations, was already attained by the comparison with experimental data [43].

Relying on this comparison, we can say that, from a mathematical point of view, both computational models lead to the same results. However, the choice of a computational approach must also take into account the computational effort required to solve the equations. As reported in Table 1, using the **A**-formulation drastically reduces the computational time, although the degrees of freedom (DOF) associated with the **A**-formulation-based model are about 10 times more than those of the **H**-formulation-based model.

The solution of this benchmark problem proves that the **A**-formulation approach is the most performing in terms of computational time. Moreover, it shows the viability of this approach for the solution of shielding problems, confirming the positive outcome of the first validation procedure achieved by the comparison between computed and experimental data [43]. For this reason, we chose the **A**-formulation approach to investigate the shielding abilities of the new shield configurations addressed in the next sub-sections.

### 3.2. Tube-Shaped Shields

The analysis addressed in the previous section also evidences that the shielding ability of a short SC tube is strongly reduced already at small tilting angles of the applied field with respect to the shield axis. For this reason, we investigated a new hybrid arrangement consisting in a ferromagnetic tube superimposed to the same SC tube analysed in the previous sub-section.

To this aim, the FM shield was modeled by an **A**-formulation as well, and the magnetic properties of the FM material were defined by the interpolation of the magnetic flux density versus the applied field curve measured experimentally on a small piece of the same material [21]. The model was implemented by means of the same Magnetic Fields interface of COMSOL Multiphysics^®^ already used for the superconductor modeling.

**Figure 4:**
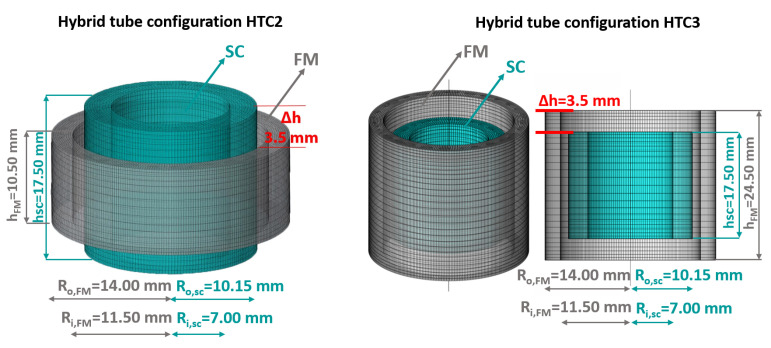
Schematic view of the tubular hybrid layouts consisting in SC/FM superimposed tubes with the SC tube protruding above the FM one (HTC2) and in SC/FM superimposed tubes with the FM tube protruding above the SC one (HTC3). The shield sizes are reported in the picture, where R_o_ and R_i_ are the outer and inner radii of the tubular layouts, respectively; Δh represents the height difference between the tubes edges.

Two different configurations were investigated, henceforth labeled hybrid tube configuration HTC2 and hybrid tube configuration HTC3, both shown in Figure 4. It is worth mentioning that we did not consider combinations of SC and FM tubes with the same height. This is due to the fact that previous investigations [60,61] highlighted that the occurrence of a shift in the edge of the two shields is a key factor to optimize the performances of the device.

**Figure 5:**
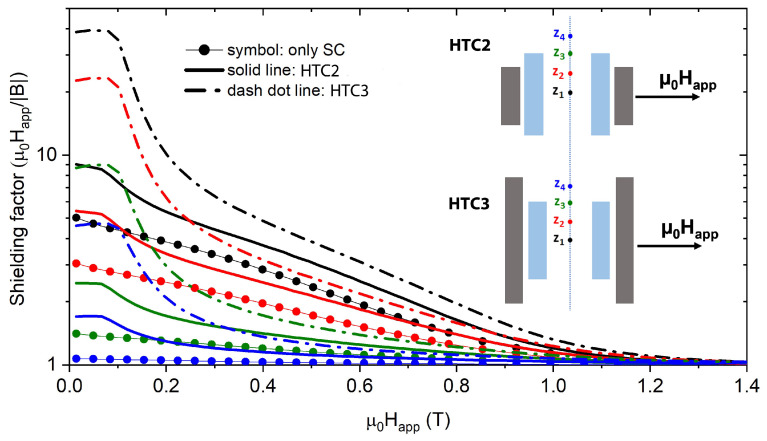
Comparison between the SFs of the single SC tube and of the HTC2 and HTC3 configurations in the TF mode. Assuming (x,y,z) = (0,0,0) the coordinate of the tube center, the plotted curves were calculated at the *z* values: $z_{1}$ = 0 mm, $z_{2}$ = 4.4 mm, $z_{3}$ = 8.8 mm (SC shield edge coordinate) and $z_{4}$ = 12 mm.

**Figure 6:**
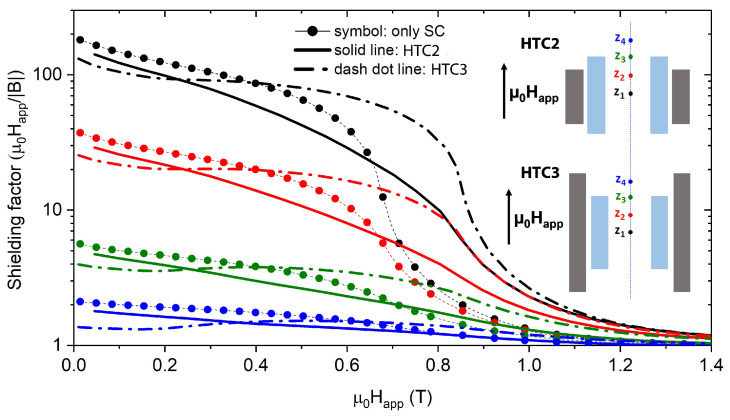
Comparison between the SFs of the single SC tube and of the configurations HTC2 and HTC3 in the AF mode. Assuming (x,y,z) = (0,0,0) the coordinate of the tube center, the plotted curves were calculated at the *z* values: $z_{1}$ = 0 mm, $z_{2}$ = 4.4 mm, $z_{3}$ = 8.8 mm (SC shield edge coordinate), and $z_{4}$ = 13.1 mm.

Figure 5 and Figure 6 shows the shielding performances of the single SC tube and of the configurations HTC2 and HTC3 for the TF and AF orientations, respectively. The comparison was carried out taking into account the shielding factor (SF) parameter, defined as $SF=\mu_{0}H_{app}/\left|B\right|$, and the ratio between the SF of hybrid shield, $SF_{hybrid}$, and that of the only SC shield, $SF_{supercond}$.

As can be seen, in the TF orientation, the configuration HTC3 shows the best shielding performances in the whole investigated range of fields. In particular, at low applied fields, an SF=40 can be reached against SF values lower than 10 obtained with the SC shield alone. This improvement is caused by the FM material property to attract the magnetic flux lines, thus reducing the magnetic flux density at the superconducting shield openings, which, due to the small aspect ratio of the tubes, affects the SF throughout the shield. Noticeably, the longer the FM tube is, the greater the improvement is, as it appears from the comparison between the HTC2 and HTC3 shielding factors.

Conversely, in the AF orientation (Figure 6), the superimposition of the FM tube induces a slight SF worsening at low applied field. Indeed, contrary to what happens in the TF orientation, in the AF orientation, the attraction of the magnetic flux lines by the FM tube increases the magnetic flux density at the SC shield openings, leading to a reduction in the shielding ability all along the shield axis. However, by raising the applied field, the flux penetration from the lateral wall becomes significant, and the screening effect by the FM tube makes the SF of the hybrid configurations overcome that of the single superconducting cup. Once again, the configuration HTC3 is the most efficient: Using this configuration, the upper limit of the region where high-SFs can be achieved is shifted from about 0.6 T to about 0.8 T.

This analysis evidenced that the positive/negative effect of the addition of the FM shell at low fields strongly depends on the field orientation and on the relative height of the FM and SC components. With the aim to deeper investigate the influence of the FM shield superimposition under realistic operating conditions, the analysis of the shielding properties was extended to magnetic fields with intermediate inclinations with respect to the shield axis, namely 7.5°, 15°, 30°, 45°, and 60°. Since, based on the previous results, the hybrid configuration HTC3 turns out to be the better performing, we focused on the comparison between HTC3 and the only SC tube. Moreover, we focused on the low applied field range (0 T $<\mu_{0}H_{app}<0.5$ T), where the actual benefit in using a hybrid configuration could be dubious.

**Figure 7:**
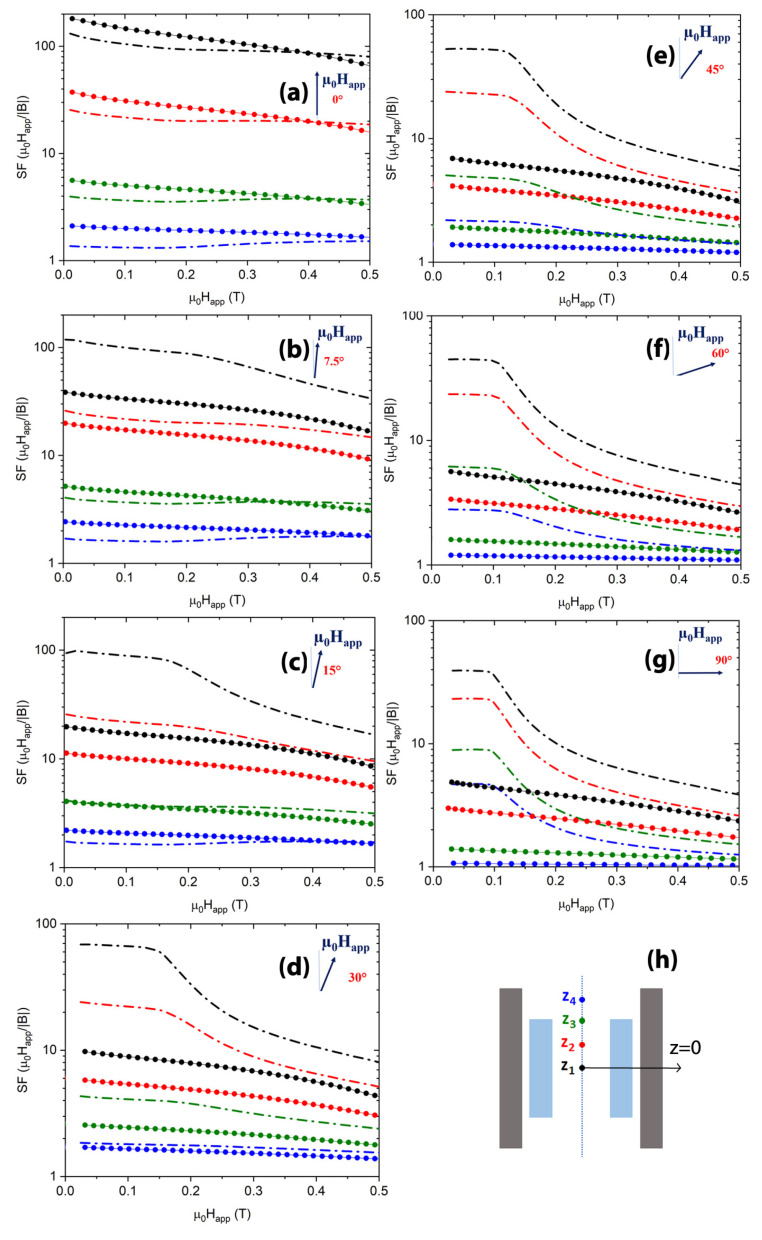
Comparison between the SFs of the single SC tube (dot symbols) and the configuration HTC3 (dash dot lines). Each frame (**a**–**g**) corresponds to a different tilt angle of the applied magnetic field. Assuming *z* = 0, the axial coordinate of the tube center, the curves refer to positions $z_{1}$ = 0 mm (black curves), $z_{2}$ = 4.4 mm (red curves), $z_{3}$ = 8.8 mm (green curves), and $z_{4}$ = 13.1 mm (blue curves), indicated in the HTC3 schematic view shown in the frame (**h**).

Figure 7 displays the SFs of the configuration HTC3 and that of the SC tube at the same positions $z_{1}$, $z_{2}$, $z_{3}$, and $z_{4}$ (Figure 7a–g). This study highlights the strong dependence of the shielding effect on the applied magnetic field orientation. Focusing on position $z_{1}$, in the range 0 T $<\mu_{0}H_{app}<0.2$ T, a field tilt of 7.5° relative to the shield axis is enough to make the hybrid arrangement 4 times more efficient than the only SC tube.

**Figure 8:**
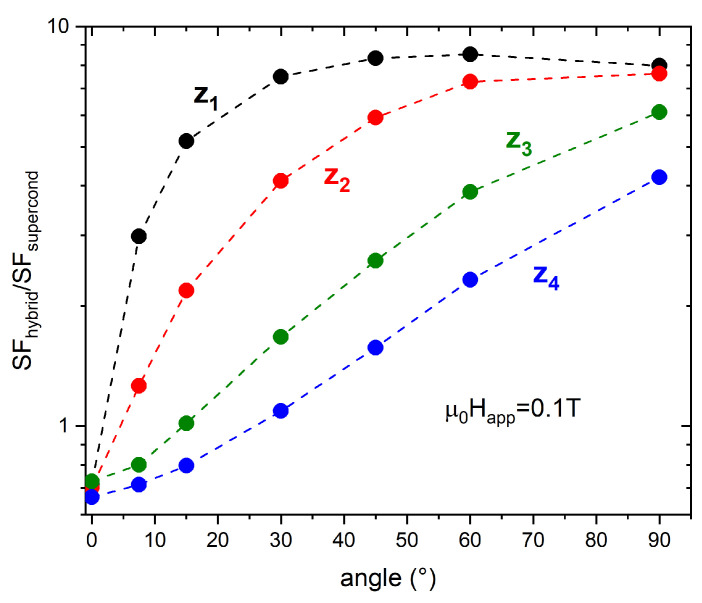
Dependence of the ratio between the SFs of the configuration HTC3 and those of the single SC tube, $SF_{hybrid}/SF_{supercond}$, on the tilt angle of the applied magnetic field having magnitude 0.1 T. The data refer to positions $z_{1}$ (black dots), $z_{2}$ (red dots), $z_{3}$ (green dots), and $z_{4}$ (blue dots).

Figure 8 sums up the ratio of the HTC3 SFs to the SC tube SFs ($SF_{hybrid}/SF_{supercond}$) as a function of the tilt angles of the applied magnetic field. At position $z_{1}$, the ratio keeps increasing with the increasing inclination of the applied field, up to reaching a saturation value of 8 for a tilt angle of 45°. A similar trend of the SF ratios can also be observed at the other positions, even though the crossover between the worsening to the improving effect of the FM shell addition is shifted to higher tilt angles of the applied field.

These results allow us to conclude that the superimposition of a ferromagnetic shield can effectively counteract the abrupt decrease in the shielding factor of a superconducting short tube occurring even for small inclination of the applied field.

### 3.3. Cup-Shaped Shields

A cap addition with a superconducting joint proved to significantly enhance the shielding ability of a short tube [11,48]. However, also in this case, the efficiency of the device results remarkably reduced when a field tilted with respect to the shield axis is applied [49,52]. Therefore, an analogous study on the effect of the superimposition of an FM shell was carried out in the case of cup-shaped shields.

**Figure 9:**
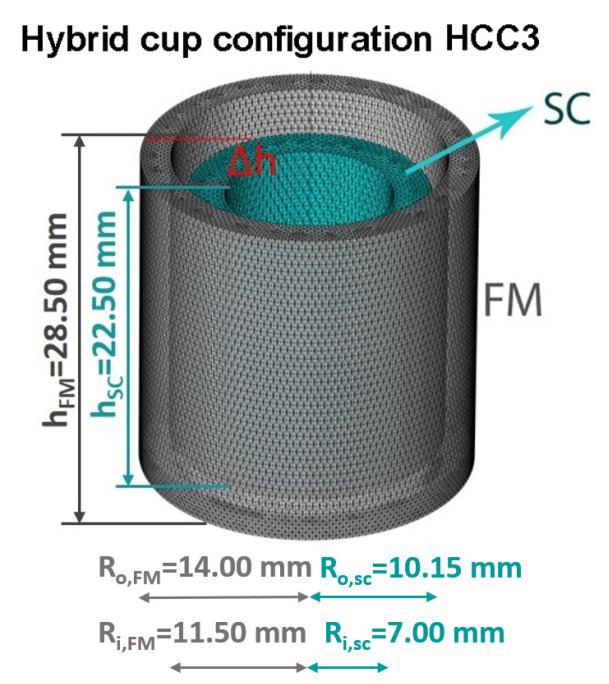
Schematic view of the hybrid layout HCC3 consisting of two SC/FM superimposed cups with the FM cup protruding above the SC one. The shield sizes are reported in the picture, where R_o_ and R_i_ are the outer and inner radii of the cup layouts, respectively; the base thickness of the SC cup is 4.20 mm, while base thickness of the FM cup is 2.50 mm; Δh=3.5 mm represents the height difference between the cup edges.

We already reported in [54] a preliminary investigation on how the SF of an SC cup is modified by superimposing a coaxial FM cup on it, as a function of the height of the FM cup. Three configurations were analyzed in the AF and TF orientations (Figure 1 in [54]). From this comparison, the hybrid cup configuration with the FM cup protruding on the SC one, henceforth labeled configuration HCC3 (Figure 9), turned out to be the highest performing, enlarging the region where shielding factors of the order of $10^{4}$ can be reached in the AF orientation up to the applied field $\mu_{0}H_{app}\approx 1.2$ T.

However, analogously to what observed for the tubular shields, at low applied fields the FM addition again worsens the shielding ability in the AF orientation, whereas improves it in the TF orientation where, near the closed extremity, the SF increases from 50 to 450 at $\mu_{0}H_{app}\approx 0.1$ T. The reason for this SF worsening/enhancement is analogous to that proposed for the hybrid tubular geometry, i.e., the presence of the FM shell causes a re-organization of the magnetic flux lines near the open edges of the HCC3 hybrid shield, hence modulating its shielding performance.

Thus, to better understand the usefulness of the FM shell superimposition, once again we extended the analysis of the shielding properties to magnetic fields with inclinations of 7.5°, 15°, 30°, 45°, and 60° with respect to the shield axis.

SC and hybrid cups’ dimensions are reported in Figure 9. To model the SC cup, we still used the approach based on the **A**-formulation, but we set the parameters in Equation (2) as $J_{c,0}=5.02\times 10^{8}$ A/m${}^{2}$, $B_{0}=0.98$ T and γ=3.78, in agreement with the experimental values attained for an MgB${}_{2}$ SC cup [54] with similar sizes.

**Figure 10:**
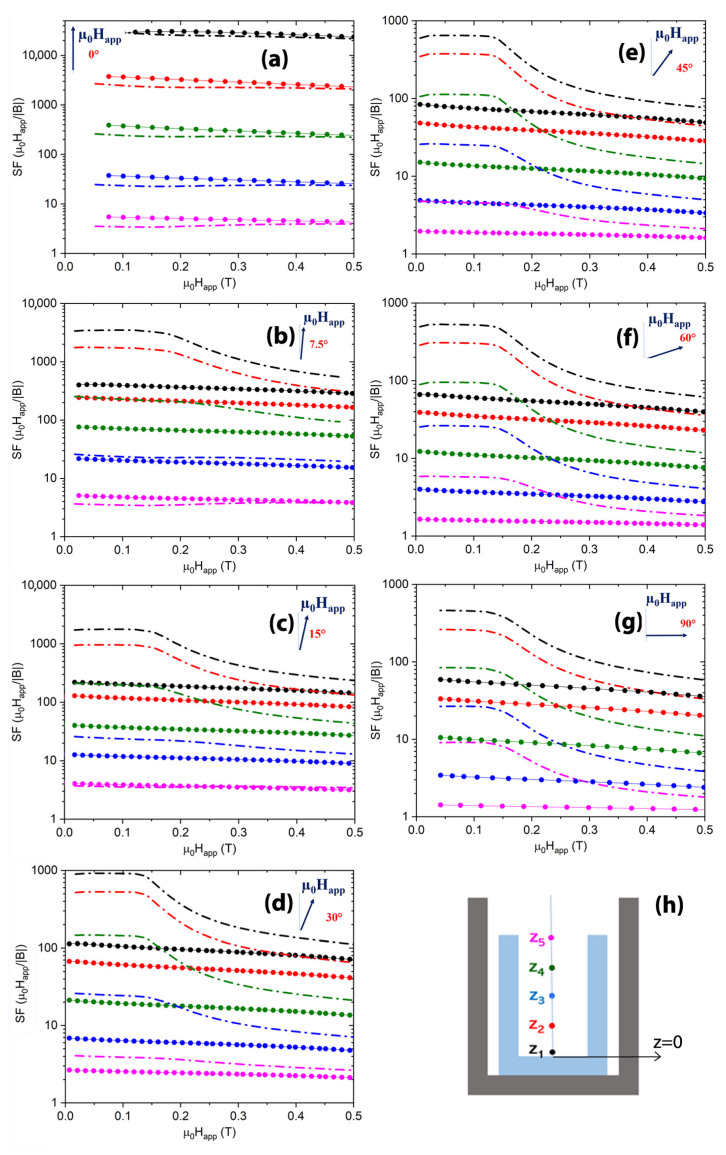
Comparison between the SFs of the single SC cup (dot symbols) and of the configuration HCC3 (dash dot lines). Each frame (**a**–**g**) corresponds to a different tilt angle of the applied magnetic field. Assuming *z* = 0 the axial coordinate of the close extremity of the SC cup, the curves refer to positions $z_{1}$ = 1.0 mm (black curves), $z_{2}$ = 5.0 mm (red curves), $z_{3}$ = 9.2 mm (green curves), $z_{4}$ = 13.7 mm (blue curves), and $z_{5}$ = 18.3 mm (magenta curves), indicated in the HCC3 schematic view shown in the frame (**h**).

Figure 10a–g show the shielding factor values of the hybrid layout HCC3 and the only SC cup at five positions along the shield axis. For the sake of consistency, the positions correspond to those selected for the comparison with the experimental data in [49]. Therefore, defining *z* = 0 to be the coordinate of the closed extremity of the SC cup, the plotted curves were calculated at the *z* values: $z_{1}$ = 1.0 mm, $z_{2}$ = 5.0 mm, $z_{3}$ = 9.2 mm (SC shield centre), $z_{4}$ = 13.7 mm, and $z_{5}$ = 18.3 mm (SC shield open extremity).

**Figure 11:**
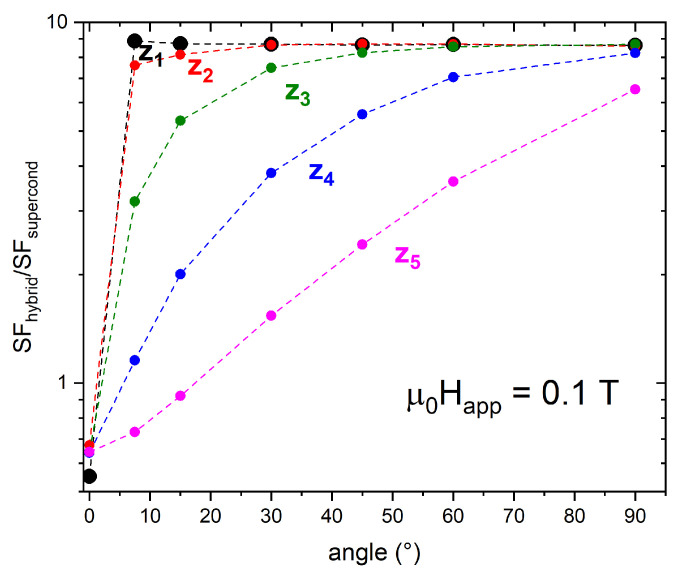
Dependence of the ratio between the SFs of the configuration HCC3 and those of the single SC cup, $SF_{hybrid}/SF_{supercond}$, on the tilt angle of the applied magnetic field having magnitude 0.1 T. The data refer to the positions $z_{1}$ (black dots), $z_{2}$ (red dots), $z_{3}$ (green dots), $z_{4}$ (blue dots), and $z_{5}$ (magenta dots).

Focusing on the inner positions $z_{1}$ and $z_{2}$, as it happened with tubular shields, in the range 0 T $<\mu_{0}H_{app}<0.2$ T, the addition of the FM cup leads to a remarkable improvement even for very small tilt angles. Indeed, in a field tilted by 7.5° with respect to the shield axis, the hybrid arrangement is ≈8 times more efficient than the only SC cup, as visible in Figure 11, where the ratio between the SFs of the configuration HCC3 and those of the single SC cup ($SF_{hybrid}/SF_{supercond}$) are plotted at $\mu_{0}H_{app}=0.1$ T. Increasing the inclination of the applied field, the SF ratio for the deepest positions keeps constant. Conversely, the SF curve ratios referring to the three outermost positions, $z_{3}$, $z_{4}$, and $z_{5}$, are smaller at low tilt angles of the applied field and show an increasing trend while increasing the field inclination. However, except for position $z_{5}$, the ratio is always greater than one, indicating a positive effect of the FM shell addition even at low tilt angles of the applied field.

## 4. Conclusions

In this paper, we investigated the shielding properties of MgB${}_{2}$ and MgB${}_{2}$/Fe hybrid shields with cylindrical symmetry. To this aim, we employed a 3D modeling approach based on the **A**-formulation, which takes considerably shorter computation time than the most commonly used **H**-formulation.

The reliability of the **A**-formulation of predicting the shielding properties of the investigated SC samples was checked comparing the data obtained solving a benchmark problem with the **A**- and the **H**-formulations. The analysis, carried out for different tilt angles of the applied magnetic field with respect to the shield axis, evidenced a noticeable agreement between the data computed with the two formulations, thus validating our 3D modeling approach.

This numerical procedure was then applied to predict the screening ability of new hybrid shield layouts consisting of coaxial open and single capped SC and FM tubes. The analysis showed that the addition of an FM shell could be a successful method to mitigate the steep reduction of the shielding capacity of short SC shields when the external field is rotated out of their symmetry axis. This effects is stronger if the open edge(s) of the FM shield protrude over the SC shield edge(s). By contrast, the FM shield superimposition induced a slight worsening in the axial field orientation. However, its effects in capped tubes (i.e., cups) can be considered negligible.

Additional refinements of the shape of both SC and FM components are still ongoing to further improve the performances of the shielding layouts. 

## Figures and Tables

**Table 1:** Comparison of the mesh degree of freedom (DOF) and computation time of the models based on the **H**- and **A**-formulations, respectively.

	DOF	Computation Time
**H**-formulation	49,479	≈120 h
**A**-formulation	405,296	≈24 h

## Data Availability

The data presented in this article will be shared on reasonable request from the corresponding author.
